# A CT-based deep learning model for preoperative prediction of spread through air spaces in clinical stage I lung adenocarcinoma

**DOI:** 10.3389/fonc.2024.1482965

**Published:** 2025-01-08

**Authors:** Xiaoling Ma, Weiheng He, Chong Chen, Fengmei Tan, Jun Chen, Lili Yang, Dazhi Chen, Liming Xia

**Affiliations:** ^1^ Medical imaging center, People’s Hospital of Ningxia Hui Autonomous Region, Yinchuan, China; ^2^ Department of Radiology, Tongji Hospital, Tongji Medical College, Huazhong University of Science and Technology, Wuhan, China; ^3^ Department of Pathology, People’s Hospital of Ningxia Hui Autonomous Region, Yinchuan, China; ^4^ Department of Radiology, Bayer Healthcare, Wuhan, China

**Keywords:** deep learning, lung adenocarcinoma, spread though air space, computer tomography, prediction

## Abstract

**Objective:**

To develop and validate a deep learning signature for noninvasive prediction of spread through air spaces (STAS) in clinical stage I lung adenocarcinoma and compare its predictive performance with conventional clinical-semantic model.

**Methods:**

A total of 513 patients with pathologically-confirmed stage I lung adenocarcinoma were retrospectively enrolled and were divided into training cohort (n = 386) and independent validation cohort (n = 127) according to different center. Clinicopathological data were collected and CT semantic features were evaluated. Multivariate logistic regression analyses were conducted to construct a clinical-semantic model predictive of STAS. The Swin Transformer architecture was adopted to develop a deep learning signature predictive of STAS. Model performance was assessed using area under the receiver operating characteristic curve (AUC), sensitivity, specificity, positive and negative predictive value, and calibration curve. AUC comparisons were performed by the DeLong test.

**Results:**

The proposed deep learning signature achieved an AUC of 0.869 (95% CI: 0.831, 0.901) in training cohort and 0.837 (95% CI: 0.831, 0.901) in validation cohort, surpassing clinical-semantic model both in training and validation cohort (all *P*<0.01). Calibration curves demonstrated good agreement between STAS predicted probabilities using deep learning signature and actual observed probabilities in both cohorts. The inclusion of all clinical-semantic risk predictors failed to show an incremental value with respect to deep learning signature.

**Conclusions:**

The proposed deep learning signature based on Swin Transformer achieved a promising performance in predicting STAS in clinical stage I lung adenocarcinoma, thereby offering information in directing surgical strategy and facilitating adjuvant therapeutic scheduling.

## Introduction

Lung cancer remains the leading lethal malignancy, responsible for 12.4% of all newly-diagnosed cases worldwide in 2022 (1). As the predominant cause of lung cancer-related mortality, lung adenocarcinoma exhibits distinctive histological growth pattern and molecular genotyping (2). Spread through air spaces (STAS) is a unique invasion pattern separate from lymphatic-vascular and visceral pleural invasion, with a predisposition in lung adenocarcinoma. Initially introduced by Kadota et al. and explicitly defined in the World Health Organization Classification of Lung Cancer in 2015, STAS refers to the dissemination of tumor cells as solid nests, micropapillary clusters or single cells into the peritumoral alveolar airspaces (3). Multiply studies have consistently demonstrated that STAS serves as a well-established prognosticator for lung adenocarcinoma undergoing sublobectomy, indicating an increased risk of postoperative relapse and worse prognosis (4–6). STAS is recognized as a pathological indicator for identifying the beneficiaries of adjuvant chemotherapy among stage IB patients (7). Therefore, STAS is of great significance in identifying high-risk patients and guiding personalized therapeutic strategies.

However, intraoperative pathological assessment for STAS through rapid frozen sections has been proved to be of limited sensitivity and reproducibility (8). The shifting of tumor cells to the peritumoral alveolar airspaces caused by manual operations such as extrusion, blade cutting and tissue dysfixation were hardly distinguished from STAS cell clusters, thereby hindering the reliable application of this approach. Several scholars exploited CT semantic indicators for STAS by visual inspection or manual measurement, such as tumor diameter, ground-glass opacity (GGO) components, and pleural retraction (9, 10). Nevertheless, these indicators rely on subjective judgement and professional skills, making them unsuitable for widespread clinical practice due to inconsistent interpretation criteria. Several studies developed CT-based radiomics signature predictive of STAS, but the radiomics approach involves several sequential processing steps such as tumor delineation, dimension reduction and model building (11, 12). The efficiency of radiomics modeling is highly influenced by interobserver heterogeneity and handling quality at each step.

Deep learning is an end-to-end network architecture, characterized by the ingestion of data from the input end and the generation of prediction results from the output end. The error between prediction result and actual observation is iteratively propagated through each layer, facilitating model adjustment and convergence. On account of the advantages of automatically learning and extracting representative information, deep learning has achieved remarkable efficacy in distinguishing histological subtypes, evaluating treatment response, and predicting survival (13–15). In this study, we employed Swin Transformer, a deep learning framework exploited by Microsoft Research Asia, to construct and validate a CT-based deep learning predictive model for STAS in lung adenocarcinoma. This study also sought to investigate the incremental value of clinical characteristics and conventional CT semantic features over the deep learning signature.

## Methods

### Patients

**Figure 1:**
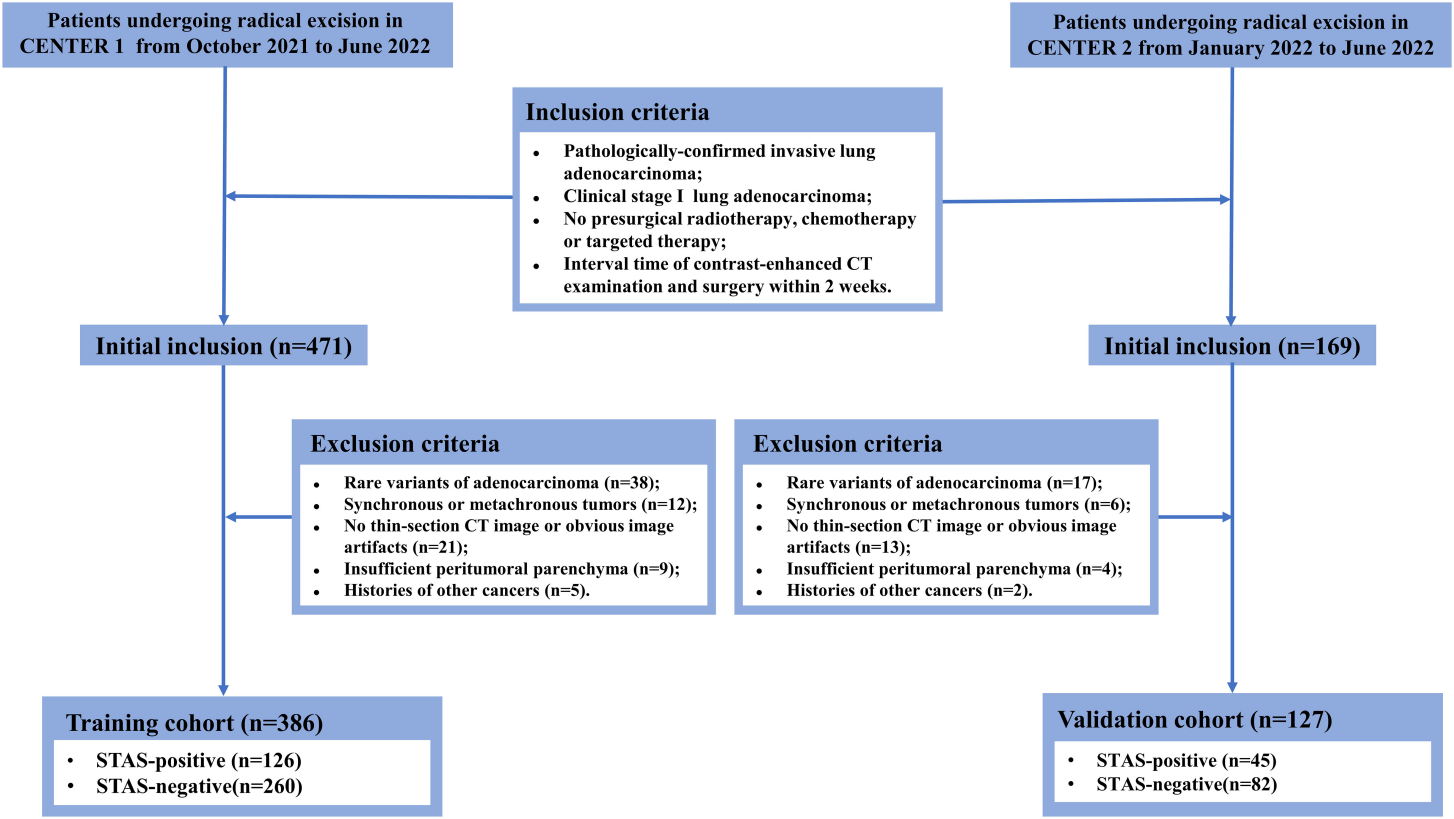
The workflow diagram of patient recruitment.

This study was approved by the Ethics Committee and the requirement for informed consent was waived due to its retrospective nature. The patients who underwent radical resection at the main campus of Tongji Hospital (Center 1) from October 2021 to June 2022 were systematically reviewed. Inclusion criteria were: (1) invasive lung adenocarcinoma confirmed by pathology; (2) maximum tumor diameter on CT images ≤ 4 cm; (3) no radiological signs of locoregional lymph node invasion or distant metastasis; (4) no preoperative radiotherapy, chemotherapy or targeted therapy; (5) interval time of preoperative CT examination and operation within two weeks. The exclusion criteria were: (a) rare histological variants; (b) simultaneous or metachronous tumors; (c) unavailable thin-section CT images or obvious image artifacts; (d) insufficient peritumoral parenchyma reserved for STAS assessment; (e) subjected to other cancers. Tumor staging was based on the eighth edition of the TNM staging system. Following the same criteria, patients undergoing radical surgical resection at the Sino-Germany Guanggu Campus of Tongji Hospital (Center 2) from January 2022 to June 2022 were retrospectively enrolled. Clinical information including gender, age, smoking history, pack-year and serum CEA level were acquired from clinical electronic records. The recruitment workflow is illustrated in **Figure 1**.

### Histological assessment

Pathological characteristics including histological subtype, Ki-67 labeling index (LI), visceral pleural invasion, lymphatic-vascular invasion, pathological TNM staging and STAS were documented. The excision specimen was fixed in 10% formalin and embedded in paraffin before sectioned. Hematoxylin-eosin staining, immunohistochemistry staining and elastic fiber staining were performed accordingly. Two pathologists with experiences of 5 years and 11 years independently interpreted STAS on the sections. Initially, tumor smooth interfaces were recognized by naked eyes and at low-magnification (×10). Subsequently, three areas with the most abundant STAS were selected for interpretation at high-magnification (×200). If any of the following forms of tumor cells are observed within peritumoral alveolar airspaces, it is judged to be STAS-positive: (1) micropapillary clusters without a central fibrovascular core; (2) solid tumor nests; (3) discrete single tumor cells. Ki-67 LI is determined by the percentage of cells with stained-brown nuclei among 1000 tumor cells via immunohistochemical staining. Invasive lung adenocarcinoma is categorized into five histological subtypes based on growth architecture: lepidic, acinar, papillary, micropapillary and solid predominant adenocarcinoma.

### CT scanning protocol and semantic feature interpretation

The patients were examined using multi-slice spiral CT scanners including GE Discovery 750 HD, TOSHIBA Aquilion One TSX-301A, Philips Brilliance ICT 256 and GE Optima CT 660. The acquisition parameters were detailed in **Supplementary Data Sheet 1**. CT semantic features were independently evaluated by two radiologists with 12 and 7 years of experience, respectively, blinded to the clinicopathological information. The lung window (width: 1600 HU; level: -600 HU) and mediastinal window (width: 400 HU; level: 40 HU) were fixed, respectively. CT semantic features included affiliated lobe, location, attenuation type, tumor total diameter, tumor consolidation diameter, consolidation-to-tumor ratio (CTR), shape, boundary, lobulation, spiculation, cavity, vacuole, air bronchogram, and plural attachment. CTR is quantified by the ratio of tumor consolidation diameter and total diameter. The definitions of CT semantic features were elucidated in **Supplementary Data Sheet 1**. The interobserver agreement for categorical and continuous variables was evaluated using Cohen ‘s kappa coefficient and intraclass correlation coefficient (ICC), respectively. The average measured by two radiologists was taken as the final value for continuous variables. Consensus on divergent categorical variables was reached through discussion involving a third radiologist.

### Tumor segmentation and deep learning signature development

**Figure 2:**
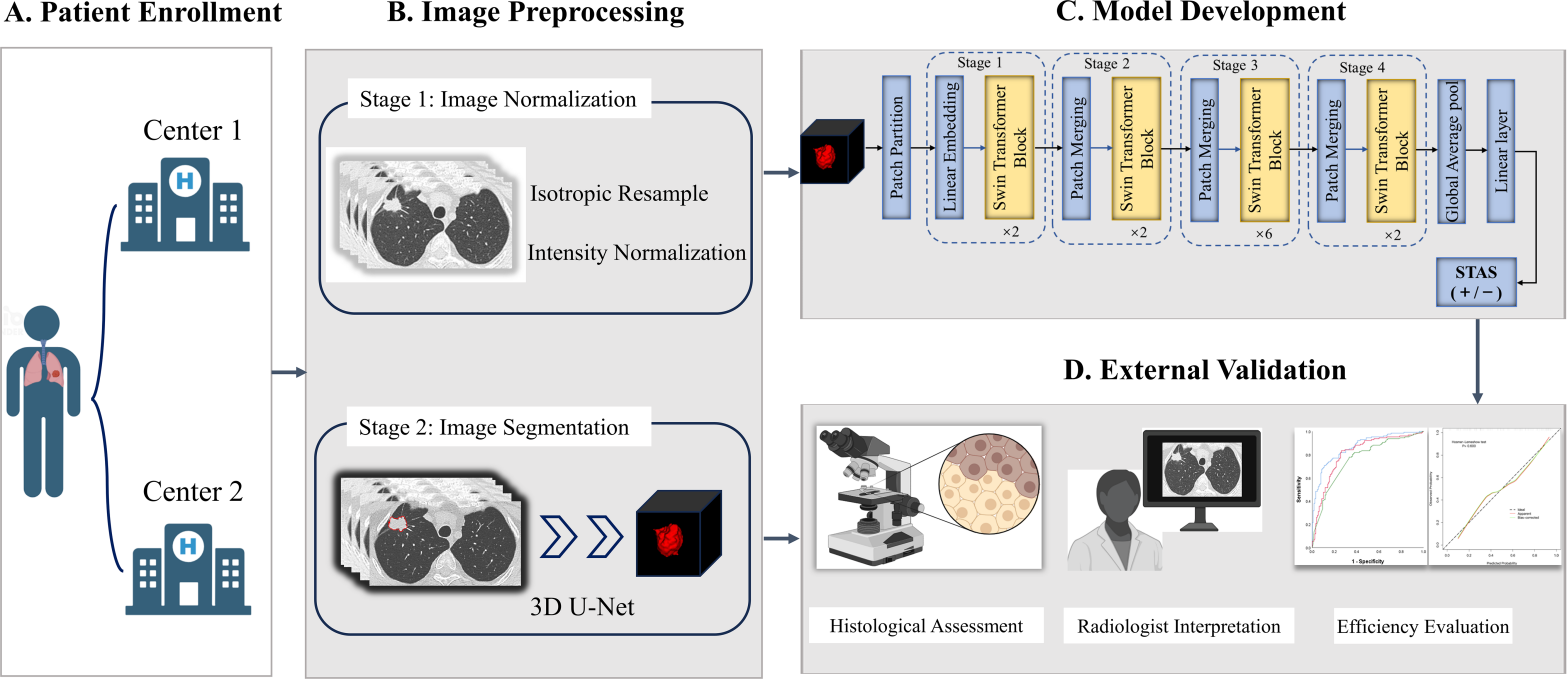
The overall framework of STAS prediction model development and validation. **(A)** Patients with lung adenocarcinoma were respectively enrolled from Center 1 and Center 2. **(B)** Imaging preprocessing included isotropic resample, intensity normalization and tumor automatic segmentation. **(C)** Deep learning signature predictive of STAS was developed based on Swin Transformer. **(D)** Histological assessment and radiologist interpretation were conducted for all patients in Center 1 and Center 2, and then model performance comparisons were performed. STAS, spread through air spaces.

The automatic virtual adversarial training segmentation algorithm, based on a three-dimensional U-shape convolutional neural network known as 3D U-Net, was employed to achieve tumor segmentation. The topology of U-net was showed in **Supplementary Data Sheet 1**. For modeling, we proposed a deep learning framework called Swin Transformer to develop a signature predictive of STAS. The overall architecture consists of four transformer stages comprising Patch Embedding/Merging and Swin Transformer Blocks in each stage as revealed in **Figure 2** and **Supplementary Data Sheet 1**. To mitigate overfitting due to limited amounts of data, the model was pretrained in CT images of lung cancer from the Cancer Imaging Archive followed by fine-tuned in 13510 CT images of lung adenocarcinoma in the training cohort. Furthermore, to compare the efficacy of different deep learning methods in predicting STAS, we applied ResNet-50, EfficientNet and ConvNeXt for modeling denoted as Model_ResNet-50_, Model_EfficientNet_ and Model_ConvNeXt_. The original code for implementing Swin Transformer can be acquired at https://github.com/microsoft/Swin-Transformer. We implemented the neural network using PyTorch 1.4.1 library in Python 3.7.0 (https://pytorch.org).

### Clinical-semantic model construction

Univariate analysis was initially performed to identify statistically significant clinical characteristics and CT semantic features between STAS positive and negative subgroups (*P* < 0.05) in the training cohort. Afterwards, features with Spearman correlation coefficient > 0.7 were removed in view of multicollinearity inference. The remaining features as candidate variables were included in multivariate logistic regression analysis to determine the features independently associated with STAS. The features were combined linearly weighted by their corresponding regression coefficients to construct clinical-semantic model. Given that the inherent design of preoperative prediction, pathological indicators were not included in logistic regression analysis, but compared across different STAS subgroups.

### Statistical analysis

Statistical analysis was performed using MATLAB (MathWorksInc., Natick, MA) and SPSS (IBM, ver.26.0). Shapiro-Wilk test and Levene test were used to analyze the normality and homogeneity of variance for continuous variables. The continuous variables were compared using the Student’s t-test and Mann-Whitney U test, as appropriate. The comparisons of categorical variables were conducted by Chi-square test or Fisher exact test. Pearson correlation analysis was used to evaluate the correlation between features. The area under receiver operating characteristic curve (AUC), sensitivity, specificity, positive predictive value (PPV) and negative predictive value (NPV) were used to quantify model performance. The calibration curve and Hosmer-Lemeshow test were employed to evaluate the consistency between predicted probabilities by deep learning signature and actual observations. A double-tailed *P*<0.05 indicated statistical significance.

## Results

### Baseline characteristics

In total, 126 eligible STAS-positive and 260 STAS-negative patients from Center 1 were enrolled to construct a training cohort (n=386). Accordingly, a total of 45 STAS-positive and 82 STAS-negative patients from Center 2 constituted an independent validation cohort (n=127). As revealed in **Table 1**, all clinicopathological characteristics and CT semantic features exhibited a balanced distribution between the training cohort and validation cohort. Of 513 patients, 239 (46.6%) were male [median age (interquartile): 59.0 (53.0, 65.0)] and 274 (53.4%) were female [median age (interquartile): 61.0 (54.0, 68.0)]. Totally, there were 171 (33.3%) and 342 (66.7%) patients pathologically-confirmed to be STAS-positive and STAS-negative, respectively.

**Table 1:** The distribution of clinicopathological characteristics in training cohort and validation cohort.

Characteristic	All patients	Training cohort	Validation cohort	*P* value
	(n=513)	(n=386)	(n=127)	
A. Clinical characteristics				
Gender				0.108
Female	274 (53.4%)	214 (55.4%)	60 (47.2%)	
Male	239 (46.6%)	172 (44.6%)	67 (52.8%)	
Age^*^ (year)	60.0 (54.0, 66.0)	59.0 (54.0, 67.0)	62.0 (53.0, 66.0)	0.320
Smoking history				0.728
Nonsmoker	369 (72.0%)	280 (72.5%)	89 (70.1%)	
Former smoker	70 (13.6%)	50 (13.0%)	20 (15.7%)	
Current smoker	74 (14.4%)	56 (14.5%)	18 (14.2%)	
Pack-year				0.907
≤ 3	372 (72.5%)	280 (72.5%)	92 (72.4%)	
4-40	89 (17.3%)	68 (17.6%)	21 (16.5%)	
> 40	52 (10.2%)	38 (9.9%)	14 (11.1%)	
CEA (ug/L)				0.930
≤ 5	435 (84.8%)	327 (84.7%)	108 (85.0%)	
> 5	78 (15.2%)	59 (15.3%)	19 (15.0%)	
Surgical modalities				0.367
Wedge resection	14 (2.7%)	12 (3.1%)	2 (1.6%)	
Sublobectomy	25 (4.9%)	21 (5.4%)	4 (3.1%)	
Lobectomy	474 (92.4%)	353 (91.5%)	121 (95.3%)	
B. Histopathological characteristics				
Histological subtype				0.352
Lepidic	88 (17.2%)	63 (16.3%)	25 (19.7%)	
Acinar	240 (46.8%)	185 (47.9%)	55 (43.3%)	
Papillary	103 (20.0%)	78 (20.2%)	25 (19.7%)	
Micropapillary	43 (8.4%)	28 (7.3%)	15 (11.8%)	
Solid	39 (7.6%)	32 (8.3%)	7 (5.5%)	
Ki-67 LI^*^ (%)	10 (3.5, 20.0)	9 (5, 20)	10 (3, 20)	0.171
Ki-67 LI				0.817
< 10%	255 (49.7%)	193 (50.0%)	62 (48.8%)	
≥ 10%	258 (50.3%)	193 (50.0%)	65 (51.2%)	
Visceral pleural invasion				0.786
Present	93 (18.1%)	71 (18.4%)	22 (17.3%)	
Absent	420 (81.9%)	315 (81.6%)	105 (82.7%)	
Lymph-vascular invasion				0.112
Present	70 (13.6%)	58 (15.0%)	12 (9.4%)	
Absent	443 (86.4%)	328 (85.0%)	115 (90.6%)	
Pathological T stage				0.176
T1a	64 (12.5%)	48 (12.4%)	16 (12.6%)	
T1b	238 (46.4%)	170 (44.1%)	68 (53.5%)	
T1c	103 (20.1%)	85 (22.0%)	18 (14.2%)	
T2	108 (21.0%)	83 (21.5%)	25 (19.7%)	
Pathological N stage				0.402
N0	437 (85.1%)	328 (85.0%)	109 (85.8%)	
N1	27 (5.3%)	23 (6.0%)	4 (3.1%)	
N2	49 (9.6%)	35 (9.0%)	14 (11.1%)	
STAS				0.563
Positive	171 (33.3%)	126 (32.6%)	45 (35.4%)	
Negative	342 (66.7%)	260 (67.4%)	82 (64.6%)	

Unless otherwise stated, data were presented as numbers (percentages) and compared using the Chi-square test or Fisher’s exact test.

*Data were presented as medians (inter-quartiles) and compared using the Mann-Whitney U test.

CEA, carcinoembryonic antigen; CTR, consolidation-to-tumor ratio; LI, labeling index; T, tumor; N, node; STAS, spread through air space.

### The interobserver consistency assessment for CT semantic features

As shown in **Table 2**, ICC for tumor total diameter, tumor consolidation diameter and CTR were 0.988 (95% CI: 0.985, 0.990), 0.991 (95% CI: 0.990, 0.993) and 0.982 (95% CI: 0.979, 0.985), respectively. Cohen ‘s kappa coefficients for the categorical variables ranged from 0.808 to 0.992, indicative of satisfactory interobserver agreement in interpreting CT semantic features. The discrepant numbers (frequency) of categorical variables between two radiologists were also documented as revealed in **Table 2**.

**Table 2:** The interobserver agreement of CT semantic features for lung adenocarcinoma.

CT semantic feature	Disagreement	Kappa value/ICC	95% CI
Affiliated lobe^‡^	2 (0.4%)	0.992	0.980, 1.000
Location^‡^	20 (4.9%)	0.808	0.728, 0.888
Tumor total diameter^§^	NA	0.988	0.985, 0.990
Tumor consolidation diameter^§^	NA	0.991	0.990, 0.993
CTR^§^	NA	0.982	0.979, 0.985
Shape^‡^	17 (3.3%)	0.883	0.826, 0.940
Boundary^‡^	29 (5.7%)	0.844	0.789, 0.890
Lobulation^‡^	9 (1.8%)	0.871	0.787, 0.955
Spiculation^‡^	12 (2.3%)	0.953	0.928, 0.978
Cavity^‡^	8 (1.6%)	0.941	0.900, 0.982
Vacuole^‡^	11 (2.1%)	0.859	0.777, 0.941
Air bronchogram^‡^	37 (7.2%)	0.856	0.811, 0.901
Pleural attachment^‡^	13 (2.5%)	0.945	0.914, 0.976

^§^ICC was calculated for the continuous variables.

^‡^Cohen’s kappa coefficient was calculated for the categorical variables.

Disagreement was presented as numbers (percentages).

ICC, intraclass correlation coefficient; CTR, consolidation-to-tumor ratio; CI, interval confidence.

NA, not applicable.

### The association of clinicopathological characteristics with STAS

As shown in **Table 3**, STAS was more likely occurred in patients with pack-year > 40 (*P*=0.002) and CEA > 5 ug/L (*P<*0.001), but it had no significant association with gender, age and smoking history. STAS was more frequently observed in micropapillary and solid predominant adenocarcinoma, but rarely occurred in lepidic predominant adenocarcinomas (*P<*0.001). Furthermore, STAS was closely related with visceral pleural invasion and lymphatic-vascular invasion (*P*<0.001 and *P<*0.001). Ki-67 LI in STAS-positive subgroup significantly exceeded that of STAS-negative subgroup (*P<*0.001). Additionally, lung adenocarcinoma with higher pathological T and N stages showed a higher prevalence of STAS (*P*<0.001 for both).

**Table 3:** The relationships of clinicopathological characteristics and CT semantic features with STAS in training cohort.

Characteristics	Training cohort	STAS positive	STAS negative	*P* value
	(n=386)	(n=126)	(n=260)	
A. Clinical characteristics				
Gender				0.201
Female	214 (55.4%)	64 (50.8%)	150 (57.7%)	
Male	172 (44.6%)	62 (49.2%)	110 (42.3%)	
Age^*^ (year)	59.0 (54.0, 67.0)	59.0 (53.8, 68.3)	59.0 (54.0, 65.0)	0.422
Smoking history				0.051
Nonsmoker	280 (72.5%)	84 (66.7%)	186 (75.4%)	
Former smoker	50 (13.0%)	24 (19.0%)	26 (10.0%)	
Current smoker	56 (14.5%)	18 (14.3%)	38 (14.6%)	
Pack-year				0.002
≤ 3	280 (72.5%)	84 (66.7%)	196 (75.4%)	
4-40	68 (17.6%)	20 (15.9%)	48 (18.5%)	
> 40	38 (9.9%)	22 (17.4%)	16 (6.1%)	
CEA				< 0.001
≤ 5 ug/L	327 (84.7%)	95 (75.4%)	232 (89.2%)	
> 5 ug/L	59 (15.3%)	31 (24.6%)	28 (10.8%)	
Surgical modalities				0.147
Wedge resection	12 (3.1%)	3 (2.4%)	9 (3.5%)	
Sublobectomy	21 (5.4%)	3 (2.4%)	18 (6.9%)	
Lobectomy	353 (91.5%)	120 (95.2%)	233 (89.6%)	
B. Histopathological characteristics				
Histological subtype				< 0.001
Lepidic	63 (16.3%)	5 (4.0%)	58 (22.3%)	
Acinar	185 (47.9%)	51 (40.5%)	134 (51.5%)	
Papillary	78 (20.2%)	24 (19.0%)	54 (20.8%)	
Micropapillary	28 (7.3%)	26 (20.6%)	2 (0.8%)	
Solid	32 (8.3%)	20 (15.9%)	12 (4.6%)	
Ki-67 LI^*^ (%)	10.0 (5.0, 20.0)	10.8 (7.4, 30.0)	5.0 (3.0, 10.0)	< 0.001
Ki-67 LI				< 0.001
< 10%	193 (50.0%)	35 (27.8%)	158 (60.8%)	
≥ 10%	193 (50.0%)	91 (72.2%)	102 (39.2%)	
Visceral pleural invasion				< 0.001
Present	71 (18.4%)	36 (28.6%)	35 (13.5%)	
Absent	315 (81.6%)	90 (71.4%)	225 (86.5%)	
Lymph-vascular invasion				< 0.001
Present	58 (15.0%)	47 (37.3%)	11 (4.2%)	
Absent	328 (85.0%)	79 (62.7%)	249 (95.8%)	
Pathological T stage				< 0.001
T1a	48 (12.4%)	10 (7.9%)	38 (14.6%)	
T1b	170 (44.1%)	42 (33.3%)	128 (49.3%)	
T1c	85 (22.0%)	29 (23.1%)	56 (21.5%)	
T2	83 (21.5%)	45 (35.7%)	38 (14.6%)	
Pathological N stage				< 0.001
N0	328 (85.0%)	82 (65.1%)	246 (94.6%)	
N1	23 (6.0%)	17 (13.5%)	6 (2.3%)	
N2	35 (9.0%)	27 (21.4%)	8 (3.1%)	
C. CT Semantic characteristics				
Affiliated lobe				0.044
Upper lobe	236 (61.1%)	68 (54.0%)	168 (64.6%)	
Middle/lower lobe	150 (38.9%)	58 (46.0%)	92 (35.4%)	
Location				0.060
Central	47 (12.2%)	21 (16.7%)	26 (10.0%)	
Peripheral	339 (87.8%)	105 (83.3%)	234 (90.0%)	
Attenuation type				< 0.001
GGO	26 (6.7%)	4 (3.2%)	22 (8.5%)	
Sub-solid	208 (53.9%)	46 (36.5%)	162 (62.3%)	
Solid	152 (39.4%)	76 (60.3%)	76 (29.2%)	
Tumor total diameter (mm)^*^	22.0 (17.0, 27.0)	25.0 (19.0, 31.0)	21.0 (16.0, 26.0)	< 0.001
Tumor consolidation diameter (mm)^*^	15.5 (10.0, 23.0)	21.0 (15.8, 28.3)	13.0 (8.0, 20.0)	< 0.001
CTR^*^ (%)	78.6 (46.5, 100.0)	100.0 (78.5,100.0)	64.1 (38.1, 100.0)	< 0.001
Shape				0.065
Round or oval	324 (83.9%)	112 (88.9%)	212 (81.5%)	
Irregular	62 (16.1%)	14 (11.1%)	48 (18.5%)	
Presence of obscure boundary	101 (26.2%)	53 (42.1%)	48 (18.5%)	< 0.001
Presence of lobulation	359 (93.0%)	121 (96.0%)	238 (91.5%)	0.105
Presence of spiculation	188 (48.7%)	76 (60.3%)	112 (43.1%)	0.001
Presence of cavity	54 (14.0%)	18 (14.3%)	36 (13.8%)	0.907
Presence of vacuole	29 (7.5%)	19 (15.1%)	10 (3.8%)	< 0.001
Presence of air bronchogram	200 (51.8%)	56 (44.4%)	144 (55.4%)	0.044
Presence of pleural attachment	118 (30.6%)	48 (38.1%)	70 (26.9%)	0.025

Unless otherwise stated, data were presented as numbers (percentages) and compared using the Chi-square test or Fisher’s exact test.

^*^Data were presented as medians (inter-quartiles) and compared using the Mann-Whitney U test.

CEA, carcinoembryonic antigen; LI, labeling index; T, tumor; N, node; GGO, ground-glass opacity; CTR, consolidation-to-tumor ratio; STAS, spread through air space.

### The association of CT semantic features with STAS

**Figure 3:**
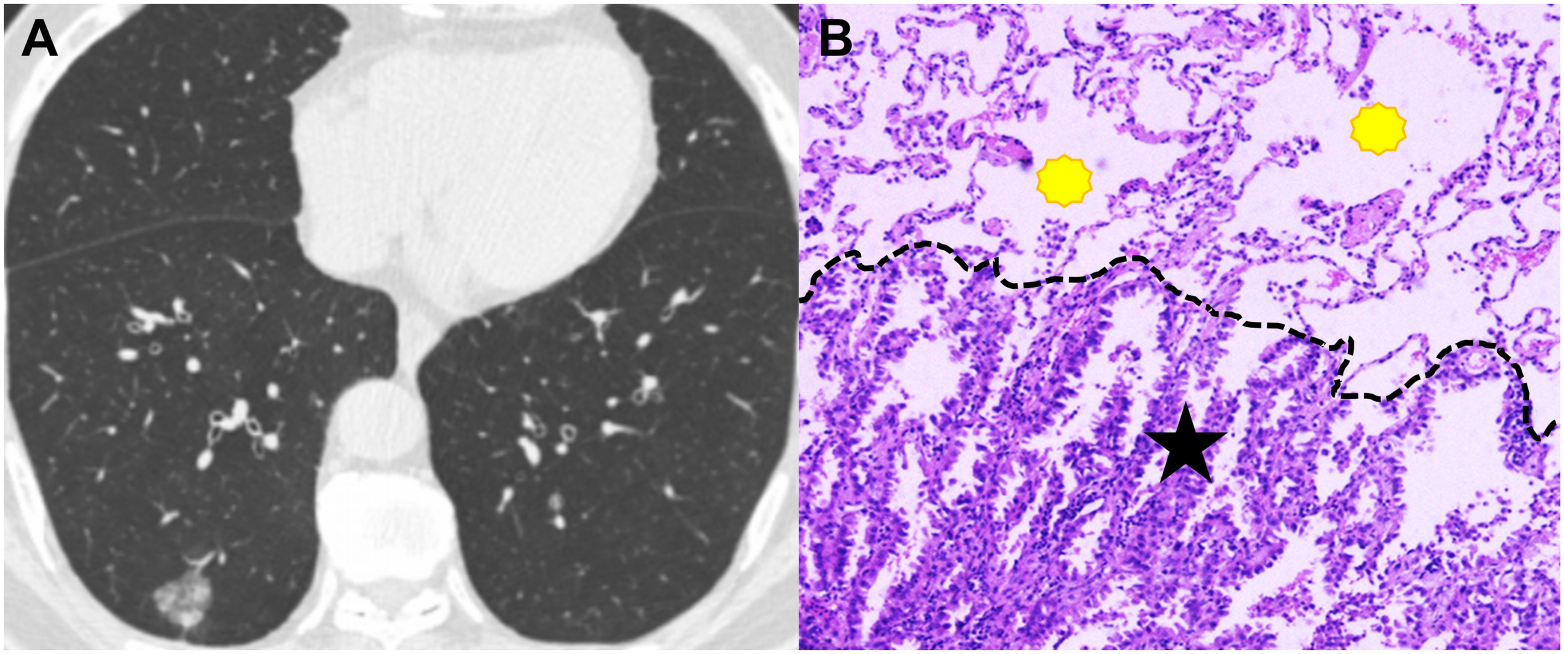
CT image and pathological image obtained from a 65-year-old man with spread though air spaces negative lung adenocarcinoma. **(A)** Th axial CT image (width, 1600 HU; level, -600 HU) shows a sub-solid nodule in the right lower lobe. **(B)** The photomicrograph of hematoxylin-eosin-stained histological section (magnification × 200) shows clean alveolar spaces (yellow polygon) beyond the boundary (dashed line) of the tumor (black star).

**Figure 4:**
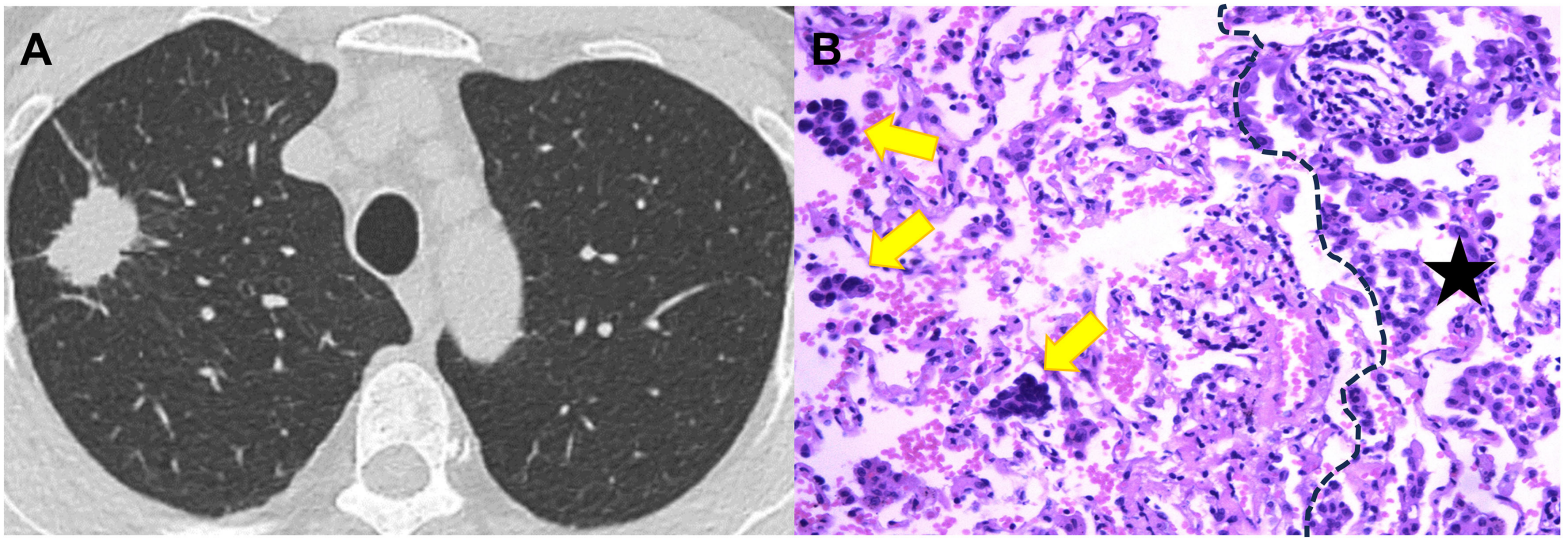
CT image and pathological image obtained from a 59-year-old woman with spread though air spaces positive lung adenocarcinoma. **(A)** Th axial CT image (width, 1600 HU; level, -600 HU) shows a solid nodule in the right upper lobe. **(B)** The photomicrograph of hematoxylin-eosin-stained histological section (magnification × 200) shows several solid nests of tumor cell (yellow arrow) beyond the boundary (dashed line) of the tumor (black star).

Tumor total diameter, tumor consolidation diameter and CTR in STAS-positive subgroup were significantly higher than those in STAS-negative subgroup (all *P*<0.001; **Figures 3** and **4**). Solid tumors, obscure boundary, spiculation, vacuole and pleural attachment were more frequent in STAS, but air bronchogram was less common in STAS (all *P*< 0.05). The tumor consolidation diameter and attenuation subtype were excluded from logistic regression analysis considering a strong correlation with CTR (r=0.839 and 0.913, *P*< 0.001). Finally, CEA (odds ratio [OR]: 2.022; 95% CI: 1.080, 3.784; *P*=0.028), vacuole (OR: 3.509; 95% CI: 1.488, 8.278; *P*=0.004), obscure boundary (OR: 2.716; 95% CI: 1.628, 4.529; *P*<0.001) and CTR (OR: 1.023; 95% CI: 1.014, 1.033; *P*<0.001) were included to construct the clinical-semantic model as the independent risk indicators for predicting STAS.

### Model construction and efficacy evaluation

**Figure 5:**
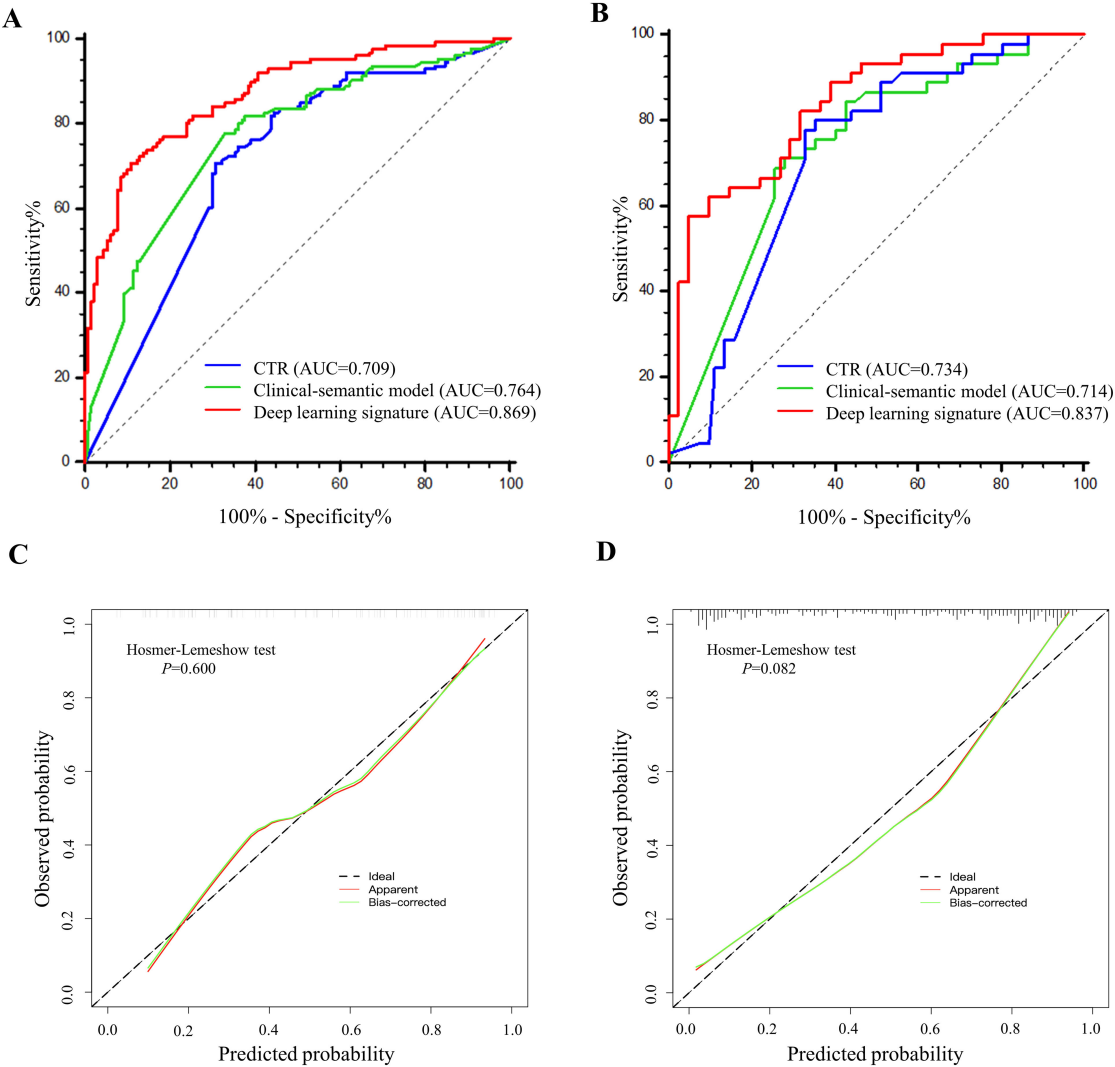
The performance comparisons of deep learning signature, CTR and clinical-semantic model in predicting STAS. **(A, B)** The receiver operating characteristic curves of CTR, clinical-semantic model and deep learning signature in training cohort **(A)** and validation cohort **(B)**. Number in parenthesis is the area under receiver operating characteristic curve. **(C, D)** The calibration curves depicted the good agreements between predicted probabilities by deep learning signature and actual observed probabilities of STAS in training cohort **(C)** and validation cohort **(D)**.

As shown in the **Table 4** and **Figure 5**, the AUC for Swin Transformer based deep learning signature in the training cohort and validation cohort was 0.869 (95% CI: 0.831, 0.901) and 0.837 (95% CI: 0.761, 0.896), respectively. Encouragingly, Swin Transformer based deep learning signature achieved significantly higher AUC than Model_ResNet-50_, Model_EfficientNet_ and Model_ConvNeXt_ in training cohort (0.869 vs. 0.800, 0.797 and 0.783; all *P* < 0.001), as well as than Model_EfficientNet_ and Model_ConvNeXt_ in validation cohort (0.837 vs. 0.775 and 0.795; *P* = 0.025 and 0.027), as shown in **Supplementary Table E2**. Deep learning signature showed an improvement in predictive performance than Model_ResNet-50_ in validation cohort, but it did not reach statistical significance (0.837 vs. 0.799, *P* = 0.087).

**Table 4:** The model performances in the training cohort and validation cohort.

Model	AUC (95% CI)	Sensitivity (95% CI)	Specificity (95% CI)	PPV (95% CI)	NPV (95% CI)
Training cohort					
CTR	0.709 (0.660,0.754)	0.706 (0.619, 0.784)	0.692 (0.632, 0.748)	0.527 (0.449, 0.604)	0.829 (0.773, 0.877)
Clinical-semantic model	0.764 (0.719,0.806)	0.778 (0.695, 0.847)	0.669 (0.608, 0.726)	0.533 (0.458, 0.606)	0.861 (0.806, 0.906)
Deep learning signature	0.869 (0.831,0.901)	0.706 (0.619, 0.784)	0.892 (0.848, 0.927)	0.761 (0.673, 0.835)	0.862 (0.815, 0.901)
Validation cohort					
CTR	0.734 (0.648,0.808)	0.689 (0.534, 0.818)	0.744 (0.636, 0.834)	0.596 (0.450, 0.731)	0.813 (0.707, 0.894)
Clinical-semantic model	0.714 (0.627,0.790)	0.778 (0.629, 0.888)	0.671 (0.558, 0.771)	0.565 (0.431, 0.691)	0.846 (0.735, 0.924)
Deep learning signature	0.837 (0.761,0.896)	0.578 (0.422, 0.723)	0.951 (0.880, 0.987)	0.867 (0.693, 0.962)	0.804 (0.711, 0.878)

CTR, consolidation-to-tumor ratio; AUC, area under the receiver operating characteristic curve; CI, confidence interval; PPV, positive predictive value; NPV, negative predictive value.

Meanwhile, The AUC for CTR alone and clinical-semantic model was 0.709 (95% CI: 0.660, 0.754) and 0.764 (95% CI: 0.719, 0.806) in training cohort, as well as 0.734 (95% CI: 0.648, 0.808) and 0.714 (95% CI: 0.627, 0.790) in validation cohort, respectively. In the training cohort, deep learning signature performed far superior to CTR (0.869 vs. 0.709, *P* < 0.001) and clinical-semantic model (0.869 vs.0.764, *P* < 0.001), with a statistically significant difference. Notably, deep learning signature yielded significantly higher AUC than both CTR (0.837 vs. 0.734, *P*=0.006) and clinical-semantics model (0.837 vs. 0.714, *P*=0.002) in validation cohort. The sensitivity, specificity, PPV and NPV of deep learning signature in predicting STAS ranged from 0.578 to 0.706, 0.892 to 0.951, 0.761 to 0.867 and 0.804 to 0.862 across two cohorts, respectively. According to the Hosmer-Lemeshow test and calibration curve, the predicted STAS probabilities by deep learning signature revealed good agreement with the actual observations both in training cohort and validation cohort (*P*=0.600 and 0.082, respectively). Furthermore, when deep learning signature was incorporated into clinical-semantic model, all CT semantic risk predictors were eliminated from multivariate regression analysis, with merely deep learning signature remained. Pearson correlation analysis revealed a strong correlation between CTR and deep learning signature (r = 0.789, *P* < 0.001).

## Discussion

This study revealed that CEA, tumor boundary, vacuolation and CTR are the independent clinical-semantic features associated with STAS in lung adenocarcinoma. The proposed deep learning model predictive of STAS based on Swin Transformer yielded an AUC of 0.869 (95% CI: 0.821, 0.908) and 0.837 (95% CI: 0.742, 0.908) in the training cohort and independent validation cohort, superior to conventional CTR and clinical-semantic model. Furthermore, neither CTR nor clinical-semantic model exhibited an incremental value over deep learning signature, further confirming its superior predictive value.

For early-stage patients, sublobectomy can preserve more pulmonary function, reduce surgical complications, and shorten hospitalization time, particularly with an equivalent therapeutic effect to lobectomy (16). However, sublobectomy is not appropriate for STAS-positive patients due to a higher risk of locoregional relapse and distant metastasis compared with lobectomy. Another study proved that STAS had negligible adverse effects on prognosis if surgical margin distance exceeded 2 cm in limited resection (17). Thus, anatomic lobectomy and sufficient surgical margin should be recommended for STAS-positive patients to prevent recurrence caused by STAS. Dai et al. also demonstrated that recurrence-free survival rates and overall survival rates of stage IA STAS-positive patients were comparable to those of stage IB patients (18). Furthermore, stage IB patients with STAS-positive can benefit from adjuvant chemotherapy (7, 19). Consequently, STAS serves as a pathological indicator for T upstaging and risk stratification, as well as an effective biomarker for identifying the beneficiaries of adjuvant chemotherapy in early-stage patients.

Currently, there is limited research on leveraging deep learning technique to predict STAS, and the predictive capacity remains modest. Tao et al. applied 3D convolutional neural network to predict STAS in NSCLC, yielding an AUC of 0.790 in validation cohort (20). Wang et al. presented SE-Resnet50 for risk estimation of STAS in solid or part-solid lung adenocarcinoma, resulting a highest AUC of 0.933 achieved so far in training cohort. Nevertheless, their model exhibited a substantial performance reduction in validation cohorts (AUC=0.783-0.806), which approximated the performance of our developed Model_ResNet-50_ in training and validation cohorts (AUC=0.799-0.800)This unfavorable generalization may attribute to model overfitting by reason of complicated architecture (21). Lin et al. enrolled 581 patients with tumor smaller than 3 cm and CTR less than 0.5 from two institutions. They extracted the deep learning features from solid components and the entire tumors respectively, thereby developing deep learning models with and without solid component gate (SCG). The results revealed deep learning model with SCG achieved higher AUCs than deep learning model without SCG (22). Thus, further investigation is required to develop deep learning signature with SCG based on Swin Transformer, in expectation to further improve the prediction efficacy. In this study, Swin Transformer was adopted as the backbone architecture in modeling, achieving a satisfactory and comparable performance in training and validation cohorts with AUC ranging from 0.837 to 0.869, which was superior to Model_ResNet-50_, Model_EfficientNet_ and Model_ConvNeXt_. This finding lent support to the potential of our proposed Swin Transformer in predicting STAS in lung adenocarcinoma. The state-of-the-art Swin Transformer is regarded as the new backbone of machine vision. With two key strengths of non-overlapping shifted windows and hierarchical structures, Swin Transformer can flexibly process images at various scales and reduce computational complexity from the exponential level to the linear level. Growing evidence validated the efficient processing capabilities of Swin Transformer in handling multitasking such as image classification and density detection (23–25). Our previously published study has affirmed the remarkable efficiency of Swin Transformer in predicting lymph node metastasis in lung adenocarcinoma (26). Aside from that, automatic tumor segmentation was conducted in this study using a 3D U-shape convolutional neural network. This deep learning architecture serves as a highly effective tool for accurate, robust, and efficient segmentation. It surpasses the time-consuming and labor-intensive manual delineation or semi-automated segmentation, as evidenced by the Dice similarity coefficients across multiple institutions (27, 28).

Further exploring the relationship between STAS and histopathological factors, micropapillary and solid predominant adenocarcinoma were more commonly observed in STAS. Our findings demonstrated a significant association between STAS and visceral pleural invasion, lympho-vascular invasion and higher pathological T stage, consistent with previous literature (29). Additionally, lymph node invasion was more frequently found in STAS-positive subgroup (34.9% vs 5.4%). In line with our results, Vaghjiani et al. also reported that STAS was an independent predictor of occult lymph node metastasis in clinical stage IA lung adenocarcinoma (30). Although the underlying mechanism of STAS remains unclear till now, it has been found that epithelial-mesenchymal transition (EMT) prominently promotes the occurrence of STAS (31). EMT is widely recognized as a biological process wherein polarized epithelial cells transform into loosely connected interstitial cells; this process is regarded as the key driver of tumor genesis, invasion and metastasis. This may account for the strong association between STAS and the aforementioned invasive histopathological factors.

In clinical-semantic model, tumor boundary, vacuolation and CTR were the independent CT semantic features in predicting STAS. As a reflection of tumor aggressiveness, CTR weighted heavily in regression analysis with a 1.25-fold increased risk of STAS for every 10% increase. In accordance with our finding, Ding et al. and Chen et al. confirmed that CTR was independently associated with STAS (32, 33). Unexpectedly, the inclusion of all clinical-semantic risk predictors failed to show an incremental value with respect to deep learning signature. We found a strong correlation between CTR and deep learning signature (r = 0.789, *P* < 0.001), which might account for this result. These findings also lead to speculation on whether deep learning signature contains biological information regarding tumor boundary and vacuolation, which should be explored by future in-depth research. We also found CTR and clinical-semantic model showed equivalent NPV and sensitivity to deep learning signature. Notably, in both training and validation cohorts, the deep learning signature exhibited far superior AUC, specificity, and PPV compared to CTR and the clinical-semantic model, which lent support to its predominant efficacy in predicting STAS.

There are some limitations to this study. First, data were retrospectively collected from different CT equipment, so heterogeneity in acquisition parameters and reconstruction protocols might be inevitable. The class-imbalance in sample should be addressed using resample techniques in the future. Second, it is necessary to expand sample size and enroll multi-institutional data to further affirm the repeatability and generalization of deep learning signature. Besides, long-term follow up and survival data should be warranted to affirm the prognostic value of STAS, as well as the relationship of deep learning signature with prognosis. Further investigation is required to enhance the biological interpretability of deep learning, which inherently possesses a black box nature, thereby facilitating its application in clinical practice. Common approaches involve employing the Grad-CAM algorithm for generating visualizations of deep learning and incorporating attentional mechanisms into deep learning networks to achieve the significance weight of diagnosis and decision-making based on attention regions. Additionally, exploring the associations between deep learning and genomics or proteomics can further improve the biological interpretability of deep learning. Last, given that biological behavior varies in different histological subtypes of lung cancer, future research needs to supplement the predictive value of the deep learning signature for STAS in other histological subtypes.

In conclusion, the proposed deep learning signature based on Swin Transformer offers a promising predictive performance for STAS in clinical stage I lung adenocarcinoma, surpassing the conventional clinical-semantic model. The end-to-end deep learning approach harbors the potential as a well-established tool for noninvasive estimation of STAS, directing surgical strategy and facilitating adjuvant therapeutic scheduling.

## Data Availability

I’m regretful that the generated dataset is inappropriate to be disclosed so far, because some work based on parts of this dataset have not yet published. Requests to access the datasets should be directed to maxiaoling0417@163.com.
