# Supplementary material for: A CT-based deep learning model for preoperative prediction of spread through air spaces in clinical stage I lung adenocarcinoma
Source: Front Oncol. 2025 Jan 8;14:1482965. doi: 10.3389/fonc.2024.1482965 (PMC11751050; doi:10.3389/fonc.2024.1482965)
Supplement: Supplementary file 1 [file Table1.docx]

| **Model** | **AUC (95% CI)** | **Sensitivity (95% CI)** | **Specificity (95% CI)** | **PPV (95% CI)** | **NPV (95% CI)** |
| --- | --- | --- | --- | --- | --- |
| **Training cohort** |  |  |  |  |  |
| Swin Transformer | 0.869 (0.831, 0.901) | 0.706 (0.619, 0.784) | 0.892 (0.848, 0.927) | 0.761 (0.673, 0.835) | 0.862 (0.815, 0.901) |
| ResNet-50 | 0.800 (0.757, 0.839) | 0.841 (0.836, 0.847) | 0.669 (0.666, 0.673) | 0.554 (0.481, 0.626) | 0.902 (0.851, 0.940) |
| EfficientNet | 0.797 (0.753, 0.836) | 0.881 (0.876, 0.886) | 0.881 (0.876, 0.886) | 0.554 (0.483, 0.624) | 0.924 (0.876, 0.958) |
| ConvNeXt | 0.783 (0.738, 0.823) | 0.690 (0.683, 0.698) | 0.777 (0.774, 0.780) | 0.603 (0.518, 0.683) | 0.842 (0.789, 0.885) |
| **Validation cohort** |  |  |  |  |  |
| Swin Transformer | 0.837 (0.761,0.896) | 0.578 (0.422, 0.723) | 0.951 (0.880, 0.987) | 0.867 (0.693, 0.962) | 0.804 (0.711, 0.878) |
| ResNet-50 | 0.799 (0.719, 0.865) | 0.533 (0.512, 0.555) | 0.976 (0.972, 0.979) | 0.926 (0.757, 0.991) | 0.800 (0.708, 0.874) |
| EfficientNet | 0.775 (0.692, 0.844) | 0.533 (0.512, 0.555) | 0.976 (0.972, 0.979) | 0.926 (0.757, 0.991) | 0.800 (0.708, 0.874) |
| ConvNeXt | 0.795 (0.714, 0.861) | 0.467 (0.445, 0.448) | 0.976 (0.972, 0.979) | 0.917 (0.730, 0.990) | 0.777 (0.684, 0.853) |

**Table E2** The model performances of different deep learning methods in the training cohort and validation cohort.

CTR, consolidation-to-tumor ratio; AUC, area under the receiver operating characteristic curve; CI, confidence interval; PPV, positive predictive value;

NPV, negative predictive value.
