# Supplementary material for: A CT-based deep learning model for preoperative prediction of spread through air spaces in clinical stage I lung adenocarcinoma
Source: Front Oncol. 2025 Jan 8;14:1482965. doi: 10.3389/fonc.2024.1482965 (PMC11751050; doi:10.3389/fonc.2024.1482965)
Supplement: Supplementary file 2 [file DataSheet1.docx]

**CT image acquisition parameters**

Tube voltage, 120 kVp; tube current, 250-300 mA; pitch, 0.813-1.500 mm; field of view, 350×350 mm; matrix, 512×512; slice thickness, 5 mm; reconstruction thickness and thickness interval, 1.00-1.25 mm. The contrast agent (Ultravist, Ioproamine, Bayer Schering Pharmaceutical AG) was injected into the right elbow vein at speed of 2.5~3.5 mL/s and dose of 1.1mL/kg followed by flushing with 20 ml saline solution. The enhanced CT images were obtained after a scanning delay of 30s. The patient assumed a head-first position while lying on their back with arms raised above their head and completed the entire lung scanning in one breath-hold after inhalation.

**The image preprocessing and deep learning model training**

The U-net topology composes of five-stage encoding and decoding subnetworks, formulating five-level mapping scales for automatic feature extraction. The encoding part consists of two convolutional layers and a maximum pooling layer in each stage, which are used to compress spatial information. In the decoding part, there are two convolutional layers and a transpose convolutional layer in each stage for virtual adversarial training feature reconstruction. Prior to being fed in, image spatial and intensity normalization was conducted to eliminate heterogeneity across multi-vendor CT scanners and different acquisition parameters. For spatial normalization, the images were first cropped to filter the void area, then resampled into a spacing of 1.52*1.52*2.74, and finally randomly cropped into 112*160*128 for data augmentation. For intensity normalization, after filtering the void area, we collected and clipped the images into their 0.05% and 99.5% intensity values and use Z-score for normalization.

The Swin Transformer Blocks are characterized as multi-head self-attention based on shifted Window, different from traditional Transformer Blocks. The segmented images from three adjacent CT slices were combined into a three-channel image, which served as the input for the deep learning model in order to generate the probability of STAS. The three-channel images were cut into non-overlapping patches and embedded in Patch Embedding. In four transformer stages, several transformer blocks with multi-head self-attention layer were applied on these patches to produce hierarchical features, which were the same as ResNet, VGGNet, and other convolutional neural networks. The global average pooling layer averaged the spatial features to reduce dimension of extracted hierarchical features. Finally, linear layer with *softmax* function generated risk probability of STAS. Specifically, to generate a robust prediction, the actual deep learning signature is obtained by means of inputting all three-channel images of each tumor and calculating the average risk probability of STAS. The code of developed Swin Transformer framework is available at <https://github.com/CharelBIT/DeepLearningRadiomicsLNMForNSCLC>.

Table E1. The definitions of CT semantic features.

| CT semantic feature | Definition |
| --- | --- |
| Location | Central tumor refers to tumor that is located within inner one-third of the lung from the midline on axial CT images. |
| Attenuation type | GGO refers to the tumor cannot conceal the pulmonary bronchial vascular structure on high-resolution CT; solid means that the bronchial vascular structures within tumor are completely covered on high-resolution CT; sub-solid refers to tumor containing both GGO and solid component. |
| Tumor total diameter | The maximum long-axis diameter of tumor on the lung window. |
| Tumor consolidation diameter | The maximum long-axis diameter of solid component on the lung window. |
| Shape | The overall shape of tumor. |
| Boundary | The delineation between the tumor margin and peritumoral lung is clear or obscure. |
| Lobulation | The arc-shape or wavelike structure on the surface of the tumor |
| Spiculation | Radial spike-like projection at the edge of the tumor. |
| Cavity | Cystic or lacunar structure inside the tumor. |
| Vacuole | Bubble-like lucency inside the tumor. |
| Air bronchogram | Air-filled bronchial structure inside the tumor. |
| Plural attachment | Tumor attached to the pleura without gap between tumor and the pleura. |
